# Examining the Influence of Re–Used Nanofiller—Pyrolyzed Montmorillonite, on the Thermal Properties of Polypropylene–Based Engineering Nanocomposites

**DOI:** 10.3390/ma12162636

**Published:** 2019-08-19

**Authors:** Tomasz M. Majka, Oskar Bartyzel, Konstantinos N. Raftopoulos, Joanna Pagacz, Krzysztof Pielichowski

**Affiliations:** 1 Department of Chemistry and Technology of Polymers, Cracow University of Technology, ul. Warszawska 24, 31-155 Kraków, Poland; 2 Institute of Ceramics and Building Materials, ul. Postępu 9, 02-676 Warszawa, Poland

**Keywords:** polypropylene, montmorillonite, nanocomposites, pyrolysis, recycling, thermal properties

## Abstract

Pyrolysis of the polypropylene/montmorillonite (PP/OMMT) nanocomposites allows for recovery of the filler that can be then re–used to produce PP/pyrolyzed MMT (PMMT) nanostructured composites. In this work, we discuss the thermal properties of PP/PMMT composites investigated by thermogravimetric analysis (TGA), differential scanning calorimetry (DSC), and dynamic mechanical analysis (DMA). It has been found that effect of PMMT (5 wt. % and 10 wt. %) on matrix thermal stability occurs at temperatures above 300 °C. Addition of 5 wt. % and 10 wt. % of PMMT into polypropylene system gave good stabilization effect, as confirmed by the overall stabilization effect (OSE) values, which increased by 4% and 7%, respectively, compared to the control sample (PP). Interestingly, the presence of 1 wt. % and 3 wt. % of pyrolyzed clay stabilizes the system better than the same concentrations of organoclay added into polypropylene melt. DSC data revealed that pyrolyzed clay has still the same tendency as organoclay to enhance formation of the α and β crystalline PP phases only. The pyrolyzed MMT causes an improvement of the modulus in the glassy as well as rubbery regions, as confirmed by DMA results.

## 1. Introduction

The homopolymer polypropylene (PP) is the main output of propylene among other derivatives, with a two–thirds consumption rate. PP is the lightest type of plastic, with a density of 0.90 g/cm^3^. PP homopolymer has a market share of 65%–75%. Branching, reinforcing and filling of PP are methods used to produce plastics with superior mechanical properties. Among different PP configurations, isotactic polypropylene (iPP) shows appropriate optical characteristics upon recycling [1].

Crystallization behavior and the crystalline morphology of polymer melt under shear flow play a primary role in the mechanical recycling of PP by processing methods [2]. The impacts of both melting temperature and shear rate on the crystallization behavior of isotactic polypropylene (iPP) melt were investigated and it was found that under static conditions, there are only random spherulite structures. Once shear is involved, the cylindrical layers appear near both surfaces of the sample, which is consistent with the skin-core structure in the injection molded parts. Meanwhile, the β–crystals can be developed and are related to the molecular orientation, depending on the applied melting temperatures and shear rates and the crystallinity of β–crystal in the pure iPP can reach 15%.

In another work Jiang et al. [3] inspired by the bamboo–like structure, proposed an efficient and simple melt sequential injection molding method to fabricate a controllable skin–core structure of iPP samples with self–reinforcement and toughness. With increases of the melt injection number, the shear layers containing shish–kebabs were progressively thickened, resulting in an effective improvement of mechanical properties.

Wang et al. applied modified injection molding technology with twice melt filling (M2) to prepare isotactic polypropylene (iPP) samples with controllable skin–core structure [4]. Compared with the conventional injection–molded samples (M1), M2 samples presented better efficiencies in reducing the creep and recovery response, and the response was strongly influenced by the shear layer thicknesses. It was also found that the creep and recovery strain reduced with decreased temperature or with applied stress. Meanwhile another important problem arose, which still needs to be solved, i.e., polymer/layered silicate nanocomposites recycling after their service–life. Significant research efforts are directed into the field of polyolefin/montmorillonite (MMT) nanocomposites [5,6,7,8,9,10,11,12,13,14,15]. Among them, polypropylene–based nanocomposites with MMT are promising engineering materials with good mechanical and barrier properties that can be easily fabricated on industrial scale by melt processing. Interestingly, the introduction of 1 wt. %–10 wt. % of MMT results in significant improvements of mechanical strength due to filler nanometric dimensions and a high aspect ratio [16].

Incorporation of MMT into polypropylene matrix leads to changes in thermal parameters of the polymer matrix, such as the glass transition temperature and the initial thermal decomposition temperature. MMT layers, due to the ‘labyrinth’ effect, limit the oxygen diffusion into the polymer bulk and hinder volatile decomposition products to escape. Moreover, the motion of macrochains in the presence of MMT layers is considerably reduced [17,18,19,20,21,22,23,24,25,26,27,28,29,30,31,32,33,34,35,36].

In an early work, Tang et al. [37] studied thermal stability and morphology of PP/clay nanocomposites. The authors showed that the kind of organophilic montmorillonite strongly influences the morphology and thermal stability of PP/clay nanocomposites. It was postulated that an observed decrease in the initial decomposition temperature T_5%_ might occur because in the nanocomposites, the intimate contact between the macromolecules and the atoms of the silicate crystalline layers is more extensive than that in a microcomposite. At the same time, there is a catalytic action by the layered silicates, which may accelerate the charring process at the beginning of the degradation.

In another experiment, Qin et al. [38] examined the thermal stability and flammability of polypropylene/montmorillonite nanocomposites, prepared by melt intercalation. They reported that the addition of MMT could considerably increase the decomposition temperature of the PP matrix and reduce the peak heat release rate (PHRR). These effects were not only due to the barrier formation, but also due to physico–chemical adsorption of the volatile degradation products on the silicates might occur. On the other hand, the TGA profiles of the composites showed that the addition of MMT could catalyze the initial decomposition of PP under oxygen.

In 2016 Kim and Lee studied the influence of interleaved films on the mechanical properties of carbon fiber fabric/polypropylene thermoplastic composites [39]. In their work several prepregs were prepared and the next set of physical properties such as thickness, density, fiber volume fraction (V_f_), and void content (V_c_), were also examined. Compared to the composite without any inserted interleaved film, as the thickness of the inserted interleaved resin film was increased, V_c_ decreased by 51.45%. However, the tensile strength decreased by 8.75%. Flexural strength increased by 3.79% and flexural modulus decreased by 15.02%. Interlaminar shear strength increased by 11.05% and impact strength increased by 15.38%.

Effect of pyrolysis temperature on biochar microstructural evolution and its influence on biochar/polypropylene composites was also checked in Elmnour’s et al. work [40]. These pyrolyzed biochars were then characterized for their carbon content, mineral compositions, chemical functionalities, and morphological structures, to help understand their physicochemical characteristics and microstructural evolution. It was revealed that the pyrolytic condition plays a key role in the formation of biochar microstructure. These biochar samples were then utilized without any further treatments/purifications for their practical application as reinforcement materials for polypropylene composites. The type of biochar was found to significantly affect the composites properties. Differences in microstructure, surface chemistry, and chemical compositions of BCs were observed to be determining factors affecting the compatibility and thermomechanical properties of resulted composites.

In another investigation, the hybridization of hemp fiber and recycled–carbon fiber in polypropylene composites was studied [41]. The effects of hybridizing hemp fiber with recycled–carbon fiber were tested to determine the trends in mechanical properties resulting from varied weight fractions. This study showcased a 10%–15% increase in tensile strength after the reinforcement of recycled–carbon fiber with hemp fiber. A 30%–35% increase was observed in the flexure strength after the reinforcement of recycled–carbon fiber with hemp fiber. Impact strength also had an increase of 35%–40% for hemp fiber reinforced recycled–carbon fiber polypropylene composite.

The influence of MMT on the PP degradation was investigated by thermogravimetric analysis (TGA) and differential scanning calorimetry (DSC) by Fitaroni et al. [42]. Although TGA analysis showed that the higher the organoclay content, the higher the temperature at which the release of volatiles takes place, the oxidation induction time (OIT) indicated a lower temperature for the onset of exothermic reactions for these materials and consequently lower thermal stability. It was postulated that the organoclay has a negative influence on the stability of the initial steps, but acts by slowing down the degradation reactions in the subsequent steps.

We have recently developed a new method of PP/MMT recycling by pyrolysis to produce non–graphitized MMT which can be then re–applied in the preparation of nanostructured polymer composites [43]. In this work we report on the influence of pyrolyzed MMT on the thermal properties of PP–based composites studied by TGA, DSC and DMA thermal analysis methods.

## 2. Experimental

### 2.1. Materials

Polypropylene (homo–polymer PP, Moplen^®^ HP500N) was purchased from Lyondell Basell Polymers (Frankfurt, Germany). Maleic anhydride grafted polypropylene used as a compatibilizer (PP–g–MA, Polybond^®^ 3200) was supplied by Chemtura Corporation (Manchester, United Kingdom). Montmorillonite modified with a quaternary ammonium salt (OMMT, Dellite^®^ 67G) was purchased from Laviosa Chimica Mineraria S.p.A. (Livorno, Italy).

Polypropylene/compatibilizer/montmorillonite nanocomposites with 3 wt % of OMMT and containing 7 wt. % of PPMA (PP/PPMA/OMMT) were obtained by melt intercalation method with the processing parameters described in our previous works [43,44]. In the preparation process, a processing line consisting of a Brabender DR20 feeder (RHL-Service, Poznań, Poland), twin screw extruder Haake Rheomex OS PTW 16/25 (RHL-Service, Poznań, Poland), cooling bath Zamak W1500 (Zamak Mercator, Skawina, Poland) and pelletizer ZamakG–16/325 (Zamak Mercator, Skawina, Poland) was used.

The pyrolysis–filler recovery process has been described in detail in references [43]. Briefly, the obtained PP/PPMA/OMMT nanocomposites were pyrolyzed in an in–house designed pyrolysis unit [44,45] with a horizontal tubular reactor. After purging with nitrogen, the reactor was heated up to 450 °C and pyrolysis was carried out for 60 min, then the temperature was raised to 500 °C and carbonization was carried out for 30 min. The obtained pyrolyzed montmorillonite (PMMT) was put in a mortar and then minced.

Next, polypropylene/compatibilizer/pyrolyzed montmorillonite (PP/PPMA/PMMT) and polypropylene/compatibilizer/organophilized montmorillonite (PP/PPMA/OMMT) nanocomposites were obtained using a Thermo Scientific HAAKE Mini CTW Compounder (RHL-Service, Poznań, Poland) at conical screw rotational speed of 150 rpm and temperature of processing 220 °C. Composition of all samples is presented in Table 1.

### 2.2. X-ray Diffraction and Scanning Electron Microscopy (SEM)

Round–shaped samples with a diameter of 20 mm and a thickness of 2 mm were prepared using the laboratory injection machine ZAMAK WT 12 (Zamak Mercator, Skawina, Poland) (processing temperature 220 °C, mold temperature 80 °C). X-ray diffraction (XRD) measurements were recorded in the 2θ range 5–40°, with a gap of 1 mm and an aperture of 3 mm, using a X-ray powder diffractometer Bruker Phaser D2 (Labsoft, Warsaw, Poland) with a Cu Kα2 (λ = 1.54 Å) source.

The fraction of β–phase in the crystalline phase of PP was calculated as:

(1) $$K=\frac{I_{300}^{\alpha }}{\left(I_{100}^{\alpha }+I_{040}^{\alpha }+I_{130}^{\alpha }+I_{300}^{\beta }\right)}$$

where I300α is the intensity of (300) plane of *α* phase, I100α is the intensity of (100) plane of *α* phase, etc.

For morphological analysis of pristine and recovered filler, as well as PP/MMT nanocomposites, a Jeol JSM–6010LA (Joel, Warsaw, Poland) scanning electron microscope was used. Pictures of gold sputtered, cryofractured samples were obtained at an accelerating voltage of 8–20 kV.

### 2.3. Thermogravimetric Analysis

Thermogravimetric analysis (TGA) was performed using a Netzsch TG209 (Netzsch, Cracow, Poland) thermogravimetric analyzer in the temperature range 30 °C–600 °C. ~5 mg samples were heated at a constant rate of 10 °C/min under air flow of 15 cm^3^/min. In each case, the measuring corundum (α–Al_2_O_3_) pan was open. The experiments were performed in triplicates and showed good reproducibility. Averaged data are presented.

### 2.4. Differential Scaning Calorimetry (DSC)

Differential scanning calorimetry (DSC) measurements were performed using a Mettler Toledo DSC823e (Mettler Toledo, Warsaw, Poland) apparatus, purged with nitrogen. The samples were first heated from −50 °C to 200 °C at 10 °C min^−1^ to erase thermal history, and then cooled to −50 °C at the same rate, and then re–heated from −50 to 200 °C at 10 °C min^−1^, and finally re–cooled to −50 °C at the same rate.

Melting (T_m_) and crystallization (T_c_) temperatures and enthalpies were determined from the first and second scan. T_m_ was considered to be the maximum of the endothermic melting peak from the heating scans and T_c_ that of the exothermic peak of the crystallization from the cooling scans. The degree of crystallinity (*X_C_*) was calculated with the total enthalpy method (Equation (2)):

(2) $$X_{C}=\frac{\Delta H_{m}-\Delta H_{c}}{\Delta H_{m}^{0}}\cdot 100\%$$

where: ∆*H_m_*—heat of melting of polymer under investigation, determined by DSC (Jg^−1^), ∆*H_c_*—heat of crystallization determined by integrating the areas (Jg^−1^) under the peaks; ΔH^0^*_m_*—heat of melting of 100% crystalline polymer (Jg^−1^) [46].

### 2.5. Dynamic Mechanical Analysis (DMA)

Dynamic mechanical analysis (DMA) was performed with a three–point bending configuration, which only required samples with small size or small mass. The specimens were measured with a Netzsch DMA 242 (Netzsch, Cracow, Poland) to obtain the profiles of storage modulus (E′) and a loss factor (tanδ) at the fixed frequencies of 1.0, 2.5, 5.0, 10.0 and 20.0 Hz and temperature ranging from −100 °C to 100 °C, at the heating rate of 2 °C/min in nitrogen atmosphere.

## 3. Results and Discussion

### 3.1. X-ray Diffraction

**Figure 1:**
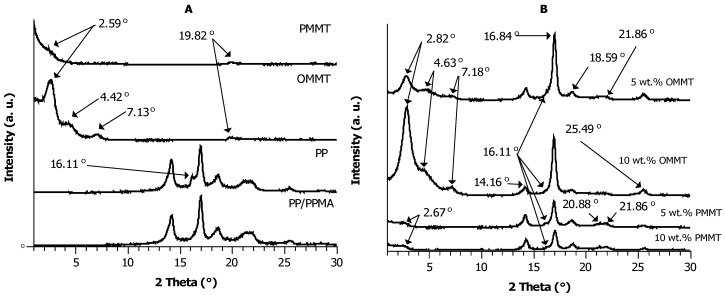
XRD (**A**) patterns of pure fillers and reference samples; (**B**) curves for polypropylene reinforced with pyrolyzed clay and control samples.

Figure 1 shows the XRD patterns of PP/PPMA/OMMT and PP/PPMA/PMMT samples with 5 and 10 wt. % of filler content. The XRD patterns of control samples of clays and neat polypropylene were presented in our earlier work [43].

Isotactic PP is a polymorphic material, which can crystallize in three different phases, namely monoclinic (α), hexagonal (β), and triclinic (γ) [47,48]. Under the processing conditions in this study, PP crystallizes mainly in the α and β forms as confirmed by the XRD reflections: The α phase is visible at 14.16° (110), 16.84° (040), and 18.59° (130), whereas the β phase shows XRD reflections at 16.19° (300), 20.88° (311), and 21.86° (131) [49]. With the addition both of higher concentration of OMMT and PMMT to PP/PPMA systems, the intensity of the reflection corresponding to the α (040) phase decreases. The recovered montmorillonite does seem to alter the crystal structure of the original polymer. The addition of 5 wt. % and 10 wt. % of OMMT and PMMT leads to nucleation of the β phase of PP. The presence of higher concentration of PMMT increases intensity reflections at 16.11°, but reduces intensity reflections in the range of 21°–22°, compared to the OMMT samples at the same concentration. However, it did not completely eliminate these reflections.

The PP/PPMA/OMMT composites containing 5 wt. % and 10 wt. % of the pristine nanofiller were characterized by three strong reflections at 2.82°, 4.63° and 7.18°, which correspond to interlayer distances of 3.13, 1.91 and 1.23 nm, respectively. Strikingly, with increasing concentration of PMMT in the composites, the intensity of its signals was reduced. In contrast, the PP/PPMA/PMMT samples with the recovered clay show only a weak signal at 2.67°, suggesting limited existence of layers with distances of the order of 3.31 nm. The negligible strength of this peak suggests that the recovered montmorillonite hardly forms any ordered structures, as actually expected also from the structure observed for the pristine filler.

**Figure 2:**
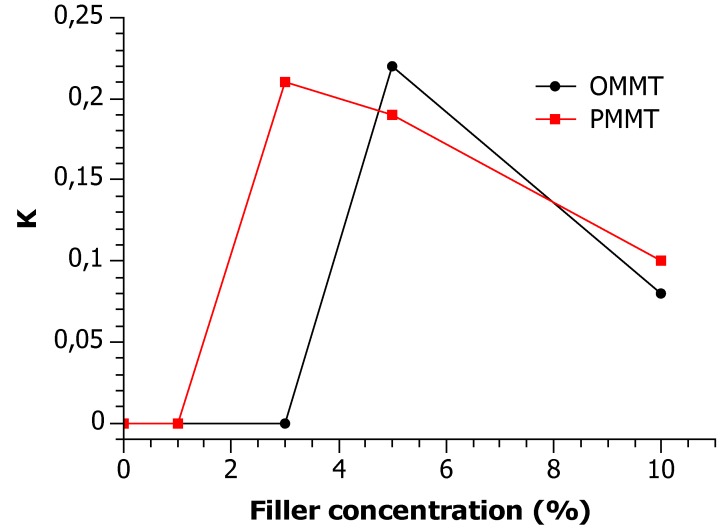
The effect of the concentration of the PMMT and OMMT filler on the average values the β–phase fraction K within the crystalline phase.

The effect of the concentration of the PMMT and OMMT fillers on the average values of the β–phase fraction K within the crystalline phase, assessed by the XRD analysis, is presented in Figure 2. Two conclusions can be derived from the presented data: First, it can be seen that the overall β–phase fraction K for PP composites decreases very slowly from 21% to 10% with the PMMT content increasing from 3 wt. % to 10 wt. %. Second, it is demonstrated that a certain critical concentration both of OMMT and PMMT is necessary to obtain a marked effect on the β–phase content.

The PP/PPMA contained α crystalline phase only, while in the neat PP profile β–phase signal was detected. When the filler concentration reached the critical value (3 wt. % for PP/PPMA/PMMT and 5 wt. % for PP/PPMA/OMMT composites), the fraction K of the β–crystallites within the crystalline phase steeply increased up to 22% and 21% for PP/PPMA/OMMT and PP/PPMA/PMMT, respectively. With a further increase of PMMT filler concentration to 10 wt. %, the fraction K of the β–phase slowly but monotonically decreased from 21% to 10%, while the results for composites including OMMT fraction K from 22% to 8%. These results show that PP–g–MA counteract nucleation of β–phase of PP, and a sufficient number of OMMT or PMMT particles is necessary for PP/PPMA systems to crystallize predominantly into β–phase. It is important that only 3 wt. % of PMMT is necessary to obtain the β–phase of PP in PP/PPMA systems, while the addition of OMMT with the same concentration did not show the same or even a similar result.

### 3.2. Scanning Electron Microscopy (SEM)

**Figure 3:**
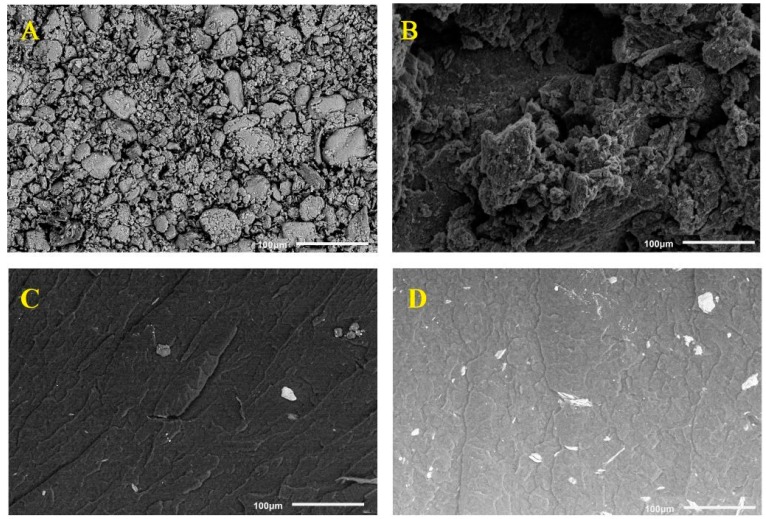
(**A**) SEM micrograph of pristine, (**B**) pyrolyzed filler, and (**C**,**D**) their 1 wt. % composites.

SEM investigations of pyrolyzed filler (Figure 3) confirm the existence of foamed, high surface area structures forming the main fraction of the pyrolysate. In both types of composites, we observe a similar degree of dispersion in the micron–scale.

Using Matlab™ software we calculated degree of dispersity on the surface of 0.17 mm^2^ for OMMT (Figure 3) and PMMT (Figure 3D) visible in images (surface of 100 × 100 μm) as light points. These backscattered SEM images of composites were chosen as representative for the whole structure because during observations very similar dispersion of particles occurred. Composites presented in the Figure 3C,D should include 1 wt. % of filler. Calculated degree of dispersity on the surface of 0.17 mm^2^ for fillers was 1.4% and 1.1%, respectively. This is evidence that despite the tendency of both fillers to agglomerate, composites with OMMT and PMMT exhibit still good dispersity after high temperature processing.

To summarize, in most cases pyrolyzed montmorillonite has a tendency to create agglomerates and aggregates of particles in the polymer melt. This suggests that the obtained polymer composites include both micro– and nanoparticles, similarly to sodium–modified MMT based polymer nanocomposites [50].

### 3.3. Thermogravimetry Analysis

**Figure 4:**
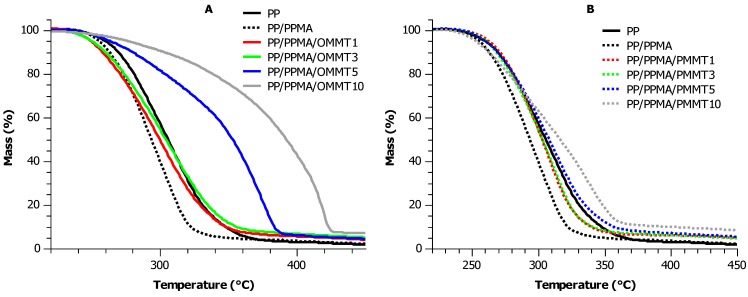
TGA profiles of (**A**) PP/PPMA/OMMT and (**B**) PP/PPMA/PMMT composites with 1 wt. %, 3 wt. %, 5 wt. % and 10 wt. % of filler.

The thermogravimetric profiles for the of PP/PPMA/OMMT and PP/PPMA/PMMT composites are presented in Figure 4A,B, respectively. The analyzed results in terms of temperatures at which 5% (T_5%_), 10% (T_10%_), 20% (T_20%_), 50% (T_50%_), and the maximum (T_max_) mass loss occurs are given in Table 2.

It can be seen from results presented in Figure 4A and Table 2 that within a temperature range 220 °C–450 °C the PP/PPMA/OMMT composite with 10 wt. % of nanofiller had the least mass loss and hence, the highest thermal stability. The thermooxidative degradation of PP/PPMA/OMMT and PP/PPMA/PMMT takes place in one step. Only the thermogravimetric curves up to ca. 480 °C of the PP/PPMA/OMMT composites containing 1 wt. % and 3 wt. % of the nanofillers are similar to these of materials with 1 wt. % and 3 wt. % of PMMT; in this temperature range one main step of degradation is observed. In the temperature range of 220–450 °C carbon–carbon bonds break, leading to the formation of unsaturated and saturated bonds along the macrochain. Addition of 5 wt. % and 10 wt. % of OMMT improved thermogravimetric factors such as the T_10%_, T_20%_ and T_max_ compared to the neat PP by 4% and 13%, 9% and 20%, 19% and 34%, respectively. At the higher temperature range, above 450 °C, stabilization action in PP/PPMA/OMMT with 5 wt. % and 10 wt. % of organoclay can be observed, which may be attributed to the ceramic–like structure formation.

The best stabilizing effect (in terms of T_50%_, T_max_, and residue values) among all the tested materials was shown by PP/PPMA/OMMT nanocomposite containing 10 wt. % of organoclay. In the range from ambient temperature to 300 °C, the TGA curves of PP, PP/PPMA/OMMT 1, PP/PPMA/OMMT 3 and PP/PPMA/PMMT 1, PP/PPMA/PMMT 3, PP/PPMA/PMMT 5 practically overlapped, suggesting within the considered temperature range a similar thermal stability. For these stabilized composites, the amount of the resulting char (at 500 °C) was approximately equal to 3%. It was also observed that the presence of PP–g–MA reduced all values of thermogravimetric coefficients compared to the neat PP.

Both OMMT and PMMT act as charring agents precursors in intumescent system. The amount of solid residue after decomposition of the PP composites including 10 wt. % of both types of fillers is about 6 wt. %. The formation of carbonaceous char residue acting as a barrier for volatile decomposition products is enhanced for PP/PPMA/OMMT and PP/PPMA/PMMT samples. It is interesting to point out that the pristine OMMT gives a better stabilization effect than PMMT. This means that pyrolyzed MMT is not as good a charring agent as organophilized clay, even though it contains small amounts of char residue particles. Nevertheless, composites containing 1 wt. % and 3 wt. % of PMMT showed much better results as compared with samples containing 1 wt. % and 3 wt. % of OMMT. This may be due to the much better affinity of PMMT to the compatibilizer, which may have a beneficial synergistic effect between components of the system at higher temperatures.

**Figure 5:**
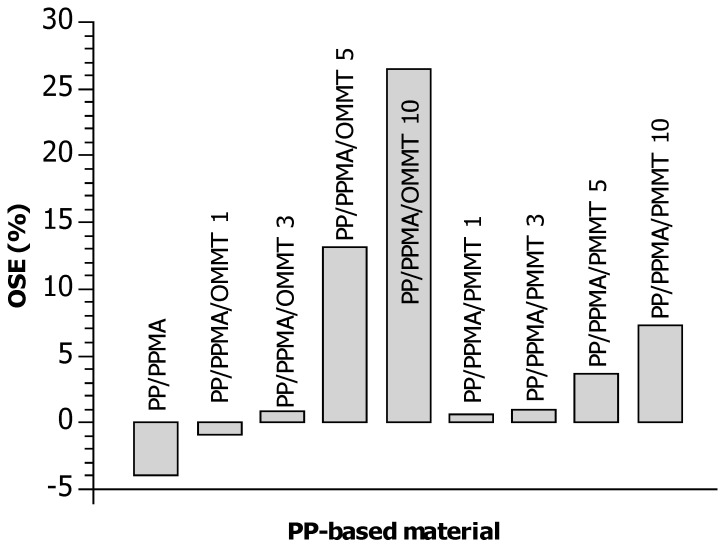
The overall thermal stabilization effect of nanofillers on polypropylene.

In order to provide a fair comparison of the effect of filler content on polypropylene, it is important to define a thermal analysis parameter that is matrix–independent. Such a parameter is called the overall stabilization effect (OSE) and it was calculated for PP/PPMA/OMMT and PP/PPMA/PMMT composites via integration of the area under the mass % versus temperature curves shown in Figure 4A,B [51]:

(3) $$OSE=\sum_{T=20}^{605}\left(\left(mass\;percent\;of\;composite_{T}\right)-\left(mass\;percent\;of\;neat\;polypropylene_{T}\right)\right)$$

where *T* is the degradation temperature. A high positive OSE value indicates an improvement in the overall thermal stability of the polymer nanocomposite in the temperature range 20 °C–605 °C, while a negative value suggests that the overall thermal stability of the composite is inferior to that of the unmodified matrix. Hence, the OSE values for PP/PPMA/OMMT and PP/PPMA/PMMT composites are presented in Figure 5.

These results are confirmation of combined actions between pyrolyzed clay or organoclay and PP–g–MA. PP with nanofillers showed mixed results; i.e., the former improved thermal stability mainly in PP/PPMA/OMMT and PP/PPMA/PMMT containing 5 wt. % and 10 wt. % of filler, but reduced the OSE value in PP/PPMA and composite with 1 wt. % of OMMT. Polypropylene with organoclay (PP/PPMA/OMMT) has the most improved thermal stability of ca. 13% and 26% for 5 wt. % and 10 wt. % of nanofiller, respectively, compared to the neat polymer. However, addition of 5 wt. % and 10 wt. % of PMMT into polypropylene system caused also a good stabilization effect confirmed by the OSE values increase by about 4% and 7%, respectively, compared to the control PP sample. It is worth pointing out that the presence of 1 wt. % and 3 wt. % of pyrolyzed clay acts better towards thermal stabilization than the same concentrations of organoclay added into polypropylene melt. These results show that PMMT can be successfully re–used as a filler and stabilizer for polypropylene in engineering applications.

Although it might be considered that the stabilizing effect of MMT has been maintained, a decrease in stability of composites with increasing PMMT content may be explained taking into account different effects. Firstly, pristine MMT can lose up to 48% by weight on ignition, while the pyrolyzed filler is far more abundant in the mineral. This could effectively mean that the MMT might not be fully sealed in carbonaceous phase of char and might act as a degradation catalyst. On the other hand, char is physically adsorbed on the surface instead and therefore might have been detached from it during processing, thereby also resulting in unprotected MMT surfaces. Another reason for this might be the presence of ligands at char surface, such as OH– or CH_3_– promoting the degradation.

### 3.4. Different Scanning Calorimetry (DSC)

In order to ensure the same thermal history for all materials, we performed two consecutive DSC heating scans, followed by cooling scans at the same rate. The heat flow curves show two distinct regions: a glass transition step and peaks related to melting/crystallization. In the following section, we will discuss both phenomena, starting with the crystallinity–related features.

**Figure 6:**
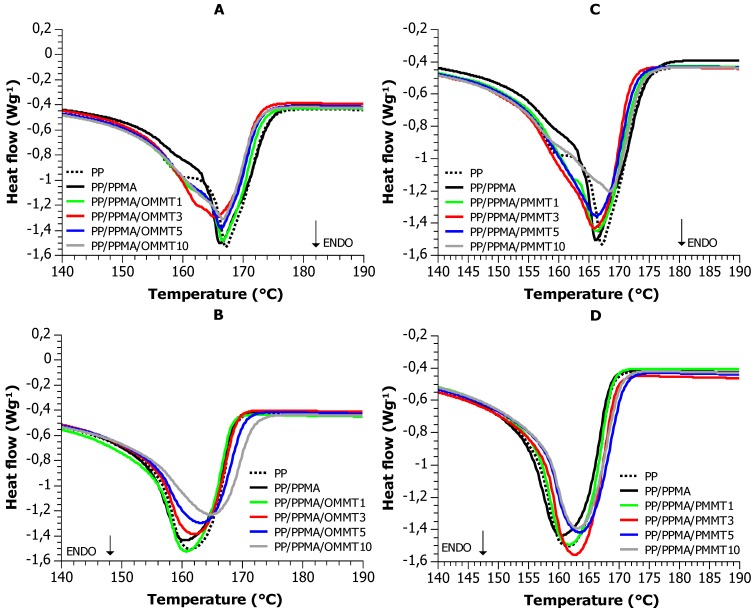
(**A**,**C**) DSC curves of the first heating and (**B**,**D**) the second heating for (**A**,**B**) PP/PPMA/OMMT and (**C**,**D**) PP/PPMA/PMMT nanocomposites.

Figure 6A,C show the melting endotherms recorded during the first run, hence probing the crystallinity of the as received materials. The melting peaks are complex with at least two melting components, around 160 °C and 168 °C, presumably corresponding to different crystallite polymorphs. Interestingly both pristine and pyrolyzed clay, seem to facilitate the formation of the low temperature polymorph, as the peak in composites is more distinct.

Contrary to the first run, the crystallization and melting endotherms at the second run (Figure 6B–D) are quite simple and no pronounced polymorphism is observed. In the following, we will discuss crystallinity based only on these runs.

The crystallization temperatures T_c_ of PP/PPMA/OMMT composites were stable for most of materials and independent from OMMT content. The T_c_ results for PP/PPMA/PMMT composites show that with the increasing content of pyrolyzed clay, the crystallization temperature shows an increasing trend, but only a moderate one and has an exception for the 10 wt. % composite. Pyrolyzed montmorillonite at lower contents probably acts as a nucleation agent during PP crystallization, whereby hindering effects occur at the highest possible content. The changes in melting and crystallization enthalpies of PP/PPMA composites confirm the thermal transformation of the components during crystallization, as well as the PP, PP–g–MA and filler interaction in creating the supermolecular structure of the PP composites (Figure 6).

The melting temperatures of PP/PPMA/OMMT and PP/PPMA/PMMT composites (Table 3) reveal the individual behaviors of the polymer component in the systems. The experimental melting temperatures of the polymeric component in the composites are comparable with the melting temperatures of homopolymers. Nevertheless, an increase of the melting temperature with increasing of both fillers content suggests that in the presence of the fillers, the resulting crystals are more stable and larger. The degree of crystallinity (Table 3) is not significantly affected by the montmorilonite, and any differences lie within the experimental error (±2%). This suggests that the filler does not change the final degree of crystallinity, although it does have a moderate effect on the dynamics of crystallization and the quality of the produced crystals, as judged by the changes in the cooling and melting temperature, respectively.

The melting enthalpies of PP/PPMA/OMMT and PP/PPMA/PMMT composites are proportional to the concentration of PP. However, two different (and reverse) trends were observed when the concentration of polymer matrix was decreased from 100 wt. % to 92 wt. %: values of melting enthalpies were first sharply reduced and then were slightly increased with further reduction of PP concentration in compositions. However, the opposite situation has been observed for samples with pyrolyzed clay. It could be explained by the effect of heating of the composite from the inside by carbonaceous char. With the increase of carbonaceous char in the composite, less energy is needed to melt the nanomaterial. This could be a critical point in the processing of PP/PMMT nanocomposites.

Glass transition is quite weak as the result of the high degree of crystallinity. Nevertheless, a moderate decrease of the T_g_ was observed for the composites with the pristine montmorillonite. This is typically attributed to increased mobility due to free volume introduced in the polymer matrix by the clay particles. This seems to not be the case for the pyrolyzed clay: the glass transition temperature remains largely unaffected, either because the clay forms larger aggregates and thus does not interact much with the matrix, or due to the counterbalancing of the free volume by increased matrix–clay interactions.

### 3.5. Dynamic Mechanical Analysis (DMA)

**Figure 7:**
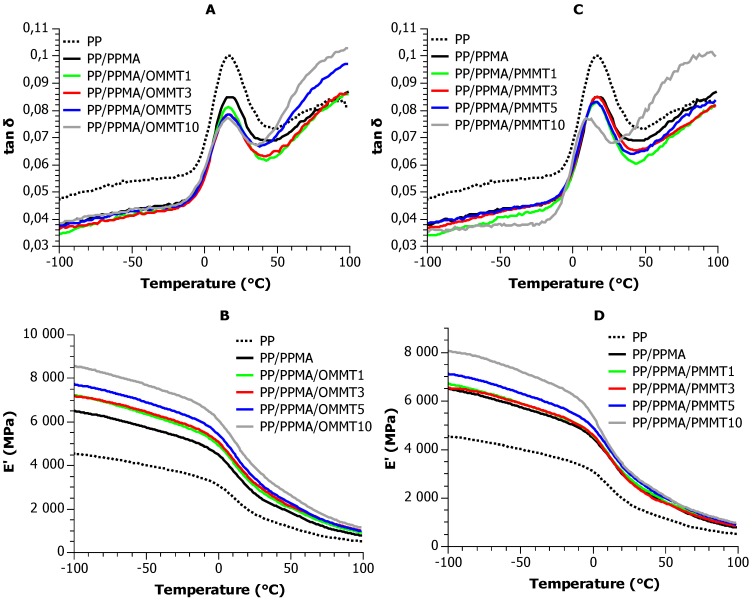
Representative thermograms (20 Hz) of the complex modulus, recorded with the matrices and selected composites: (**A**,**C**) tanδ curves vs temperature for PP/PPMA/OMMT and PP/PPMA/PMMT composites, respectively, (**B**,**D**) E′ profiles vs temperature for PP/PPMA/OMMT and PP/PPMA/PMMT composites, respectively.

Representative thermograms of the complex modulus, recorded for the matrices and selected nanocomposites, are shown in Figure 7.

Starting from low temperatures, at the region of the glassy plateau of storage modulus (E′), a very weak relaxation γ is visible as a broad peak at ca. −50 °C in loss factor (tan δ) and loss modulus (E″) curves. It has been attributed to the motion of very short segments of the macrochain [52,53]. As this relaxation is of no interest in our study, we will not comment on it further. Above 0 °C a sharper peak at E″ and tan δ accompanied by a steep step in E′ corresponds to the dynamic glass transition. Contrary to nomenclature habits dictating that such a relaxation is termed as α, for polypropylene, traditionally, it is named as β [54] and we will comply with this convention.

With a further increase in temperature, around 50 °C a step at E′ accompanied by peaks in E″ and tanδ is named α relaxation and corresponds to the slip and rotation of the crystalline lamellae [54]. Interestingly, this relaxation is enhanced in the presence of the pristine clay. This suggests that probably the compatibilized and non–carbonized clay layers have an effect similar to that of the crystalline lamellae.

In the following we will comment on the effect of the fillers on the rubbery and glassy moduli, the dynamic glass transition.

**Figure 8:**
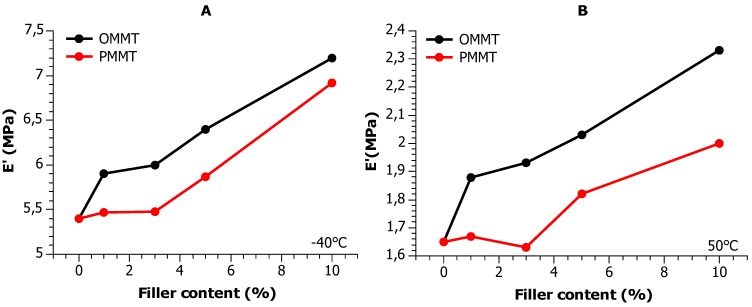
Glassy and rubbery moduli vs filler content at representative temperatures indicated on the plot.

Both the pristine and pyrolyzed filler result in improvement of the modulus in the glassy and the rubbery regions (Figure 7 and Figure 8). Interestingly, the glassy modulus improves already at 1 wt. % with the pristine nanofiller, but at least 3 wt. % of the pyrolyzed filler is needed before any significant increase of modulus is observed. 5 wt. % of the recycled filler has the same effect as 1 wt. % of the pristine, but then, in both the glassy and the rubbery moduli, the E′ vs wt. % slopes are the same. It seems that the first ca. 3 wt. % of the pyrolyzed MMT do not contribute to the mechanical properties, but then it has similar effects to the pristine filler.

**Figure 9:**
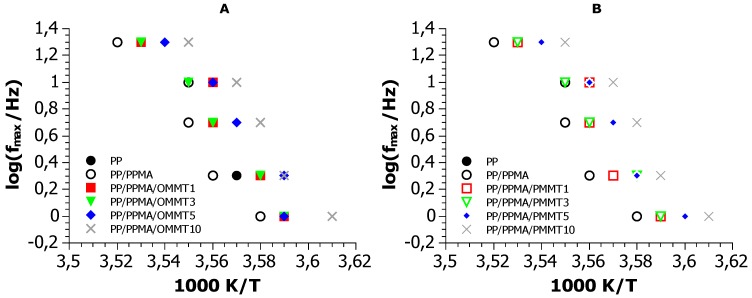
The Arrhenius plot of the dynamic glass transition peak (β) for: (**A**) samples with OMMT, (**B**) samples with PMMT.

Results on dynamics in polymers are typically presented in the so called Arrhenius plot, i.e., the logarithm of the time scale of the relaxation–usually in terms of frequency–as a function of the inverse frequency [55]. In this representation, local relaxations have linear traces following simple Arrhenius dynamics, whereas cooperative phenomena, such as the glass transition, show a concave form, described by the mostly empirical Vogel–Fulcher–Tammann model. Figure 9 shows the Arrhenius plot of the dynamic glass transition peak (β). In agreement with DSC, we observe that the compatibilizer decelerates the dynamics, acting as a plasticizer. Further, addition of filler accelerates dynamics in a monotonous manner, presumably due to introduction of free volume in the system. The effect of the two fillers is similar.

Due to the limited number of points, and the narrow frequency range of DMA, it would not be possible to observe the concavity of the relaxatrion or to calculate the dynamic parameters. However, we would like to point out that it is obvious that at least in the narrow region, a few tens of degrees above glass transition, the local activation energy, as expressed by the slope of the traces, does not seem to change neither with addition of a compatibilizer nor with an increasing filler content.

## 4. Conclusions

WAXD results confirm the persistence of the layered pyrolyzed clay structure with a higher interlayer distance than the original clay. However, with the increasing concentration of PMMT in the composites, the intensity of these signals was reduced.

From the SEM analysis it can be seen that the foamed, high area structures creating main fraction of the pyrolysate are formed. The micrograph of PMMT shows less aggregates compared to pristine OMMT. The entire surface of PMMT is covered with a thin carbon layer.

TGA profiles containing PMMT filler have very similar shape compared to the control samples (PP and PP/PPMA). The main difference between samples containing OMMT and PMMT is that 5 wt. % and 10 wt. % of OMMT improved thermal properties in the whole range of the thermal test, while stabilizing effect of 5 wt. % and 10 wt. % of PMMT is visible only above 300 °C i.e., at the later stages of decomposition. Composites containing 1 wt. % and 3 wt. % of PMMT showed much better results compared with samples contain 1 wt. % and 3 wt. % of OMMT. The values of the overall stabilization effect (OSE) parameter showed that PP with nanofillers gave mixed results; i.e., the former improved thermal stability mainly in PP/PPMA/OMMT and PP/PPMA/PMMT with 5 wt. % and 10 wt. % of filler but reduced the OSE value in PP/PPMA and sample with 1 wt. % of OMMT. Addition of 5 wt. % and 10 wt. % of PMMT into polypropylene system caused good stabilization effect confirmed by the OSE values increase by about 4% and 7%, respectively, as compared to the control sample (PP).

In the DSC experiment, during the first heating process, the shape of the main peak had a sharp nature, with maximum at constant temperature of 167 °C. For neat PP and samples containing 1 wt. % of OMMT and PMMT, the second peak has been observed at 162 °C. After re–crystallization process only one wide peak has been observed in the range of 161 °C–165 °C. Pyrolyzed clay have still the same tendency such as organoclay to influence on formation the α and β crystalline phases of PP only.

During DMA investigations, three relaxations have been observed: γ in the range of −100 °C–(−20) °C, β in the range of −20 °C–50 °C and α above 50 °C. Both the pristine and pyrolyzed filler cause an improvement of the modulus in the glassy and the rubbery regions in E′ vs filler content profiles. The glassy modulus improves already at 1 wt. % with the pristine nanofiller, but at least 3 wt. % of the pyrolyzed filler is needed before any significant increase of modulus is observed.

## Figures and Tables

**Table 1:** Composition of the PP/PPMA/OMMT and PP/PPMA/PMMT composites.

Name of Sample	Matrix (wt. %)	Compatibilizer (wt. %)	Filler (wt. %)
PP	100.0	–	–
PP/PPMA	95.5	4.5	–
PP/PPMA/OMMT	97.5	1.5	1
PP/PPMA/OMMT	92.5	4.5	3
PP/PPMA/OMMT	87.5	7.5	5
PP/PPMA/OMMT	75.0	15.0	10
PP/PPMA/PMMT	97.5	1.5	1
PP/PPMA/PMMT	92.5	4.5	3
PP/PPMA/PMMT	87.5	7.5	5
PP/PPMA/PMMT	75.0	15.0	10

**Table 2:** Temperatures at which 5%, 10%, 20% and 50% mass loss occurs for PP/PPMA/OMMT and PP/PPMA/PMMT composites.

Sample	T_5%_ (°C)	T_10%_ (°C)	T_20%_ (°C)	T_50%_ (°C)	T_max_ (°C)	Residue at 500 °C (%)
PP	260	269	280	305	314	0.2
PP/PPMA	255	263	273	294	288	1.1
PP/PPMA/OMMT 1	250	258	271	300	295	1.6
PP/PPMA/OMMT 3	250	260	273	304	307	3.2
PP/PPMA/OMMT 5	264	280	304	353	373	3.0
PP/PPMA/OMMT 10	279	304	336	393	420	5.9
PP/PPMA/PMMT 1	264	272	281	303	311	3.2
PP/PPMA/PMMT 3	262	269	279	303	312	3.0
PP/PPMA/PMMT 5	260	269	281	308	313	2.9
PP/PPMA/PMMT 10	253	262	278	316	340	6.4

**Table 3:** Glass transition temperature T_g_, and the corresponding heat capacity step ∆C_p_, melting and crystallization temperatures T_m_ and T_c_, along with the corresponding enthalpies ΔH_m_ and ΔH_c_ and degree of crystallinity X_c_. All parameters were calculated from the second heating/cooling scan.

Sample	Content of Filler (wt %)	T_g_ (°C)	∆C_p_ (J/gK)	T_m_ (°C)	ΔH_m_ (J/g)	T_c_ (°C)	ΔH_c_ (J/g)	Χ_c_ (%)
PP	–––	−13	0.12	162	56.92	116	54.97	54
PP/PPMA	–––	−13	0.14	161	55.57	116	52.29	52
PP/PPMA/OMMT	1	−15	0.13	161	58.06	116	53.73	54
PP/PPMA/OMMT	3	−17	0.12	162	54.03	116	53.10	52
PP/PPMA/OMMT	5	−11	0.06	163	55.07	116	53.17	52
PP/PPMA/OMMT	10	−24	0.10	165	56.19	114	52.69	53
PP/PPMA/PMMT	1	−12	0.10	161	54.93	118	52.37	52
PP/PPMA/PMMT	3	−12	0.15	163	58.82	118	52.80	54
PP/PPMA/PMMT	5	−14	0.10	164	56.54	119	53.33	53
PP/PPMA/PMMT	10	−15	0.12	164	54.85	120	51.42	51
